# Antifungal Mechanism of Metabolites from Newly Isolated *Streptomyces* sp. Y1-14 against Banana Fusarium Wilt Disease Using Metabolomics

**DOI:** 10.3390/jof8121291

**Published:** 2022-12-09

**Authors:** Miaomiao Cao, Qifeng Cheng, Bingyu Cai, Yufeng Chen, Yongzan Wei, Dengfeng Qi, Yuqi Li, Liu Yan, Xiaojuan Li, Weiqiang Long, Qiao Liu, Jianghui Xie, Wei Wang

**Affiliations:** 1 College of Horticulture/College of Tropical Crops, Hainan University, Haikou 570228, China; 2 Key Laboratory of Biology and Genetic Resources of Tropical Crops, Ministry of Agriculture, Institute of Tropical Bioscience and Biotechnology, Chinese Academy of Tropical Agricultural Sciences, Haikou 571101, China; 3 College of Horticulture, China Agricultural University, Beijing 100083, China; 4 College of Horticulture and Forestry Sciences/Faculty of Plant Science and Technology, Huazhong Agricultural University, Wuhan 430070, China

**Keywords:** *Streptomyces* sp., banana Fusarium wilt, antifungal mechanism, GC-MS, metabolomics

## Abstract

Banana Fusarium wilt caused by *Fusarium oxysporum* f. sp. *cubense* tropical race 4 (Foc TR4) is one of the most destructive banana diseases in the world, which limits the development of the banana industry. Compared with traditional physical and chemical practices, biological control becomes a promising safe and efficient strategy. In this study, strain Y1-14 with strong antagonistic activity against Foc TR4 was isolated from the rhizosphere soil of a banana plantation, where no disease symptom was detected for more than ten years. The strain was identified as *Streptomyces* according to the morphological, physiological, and biochemical characteristics and the phylogenetic tree of 16S rRNA. *Streptomyces* sp. Y1-14 also showed a broad-spectrum antifungal activity against the selected 12 plant pathogenic fungi. Its extracts inhibited the growth and spore germination of Foc TR4 by destroying the integrity of the cell membrane and the ultrastructure of mycelia. Twenty-three compounds were identified by gas chromatography–mass spectrometry (GC-MS). The antifungal mechanism was investigated further by metabolomic analysis. Strain Y1-14 extracts significantly affect the carbohydrate metabolism pathway of Foc TR4 by disrupting energy metabolism.

## 1. Introduction

Banana (*Musa* spp.) is an important fruit and staple crop [1]. Global banana production is approximately 153 million tons annually, supplying food for more than 400 million people [2]. Development of the banana industry is limited by Fusarium wilt caused by a soil-borne fungal disease *Fusarium oxyspoum* f. sp. *cubense* (Foc). Especially, Foc tropical race 4 (Foc TR4) is considered as one of the most destructive diseases and can infect almost all banana cultivars [3].

Although rotation can control the occurrence of banana wilt disease to some extent, the method is rarely used by farmers due to low economic returns [4]. The use of chemical fungicides not only has an economic cost but also causes environmental safety problems. The disease-resistant cultivar selection of banana by conventional breeding is a significant challenge because of inter-specific hybridization barriers, which prevents the transfer of desirable agronomic traits into the genus [5]. Biological control owns the characteristics of safety and high efficiency and has gradually become a promising strategy for the sustainable management of ecological and agricultural crops [6].

Functional microbes are an alternative to minimize the effects of chemical agents on environment pollution and human health. They become an important resource for biological control of Foc TR4 [7]. For example, *Trichoderma* strains can significantly inhibit the spread of banana wilt disease [8]. *Bacillus subtilis* R31 and TR21 showed beneficial effects on the control of Foc TR4 [9]. A growth promotion of *Burkholderia* sp. HQB-1 could be a promising biological agent against Foc TR4 [10].

Natural products (NPs) from different microbes have widely applied in medical and agricultural fields [11]. Actinomycetes are ubiquitous in various ecosystem. They produce several bioactive compounds with strong antimicrobial activities [12,13,14]. For example, endophytic actinomycetes isolated from tea plants (*Camellia sinensis*) showed strong antagonistic activities against both bacterial and fungal pathogens [15]. Our previous studies also showed that actinomycetes have a great potential to become biocontrol agents for managing banana Fusarium wilt disease [16,17,18]. For example, *Streptomyces* sp. H4 showed a high antifungal activity against Foc TR4 [16]. *Streptomyces* BITDG-11 obviously reduced the disease index of Foc TR4 on banana plantlets [17]. *Streptomyces* sp. WHL7 significantly inhibited the growth of pathogens and increased the resistance of banana plantlets to Foc TR4 in the pot experiment [18]. Although some actinomycetes against Foc TR4 were screened, they are rarely used in the field due to their isolation from extreme environments such as oceans. Structural characteristics of distinct microbiomes in the suppressive and conducive soils were detected after Foc TR4 infection [19]. Therefore, the isolation of functional actinomycetes from the suppressive soils is an important way to explore the antagonistic agents. In our study, to screen the biocontrol potential agents for Foc TR4, banana plantation soil without Foc TR4 disease symptoms for ten years was collected. These isolated species were identified by the physiological and biochemical characteristics and molecular method. The isolate of a novel strain Y1-14 with strong antifungal activity and broad-spectrum antifungal activity was selected for further study. The effects of its extracts on mycelial growth, morphology, and cell ultrastructure and the spore germination of Foc TR4 were also investigated. In addition, the antifungal mechanism was studied by gas chromatography–mass spectrometry (GC-MS) and metabolic analysis. Our purpose is to further develop stable, efficient, multifunctional, and antifungal actinomycetes with an application potential in the field.

## 2. Materials and Methods

### 2.1. Isolation of Actinomycetes

The soil samples were collected from the rhizosphere of a banana plantation in Xishuangbanna, Yunnan Province, China (21°10′–22°40′ N, 99°55′–101°50′ E). No disease symptoms of Foc TR4 were observed in the field for ten years.

Actinomycetes were isolated through a serial dilution method [20]. An amount of 5 grams of dried soil samples were diluted into 45 mL of distilled water. Rifampicin (50 mg/L) and nystatin (50 mg/L) were added to inhibit the growth of bacteria and fungi, respectively. The soil suspension was cultured at 200 rpm at 28 °C for 30 min. The soil suspension was then diluted from 10^−2^ to 10^−3^ fold and were spread on Gaoshi No. 1 medium (Qingdao Hope Bio-Technology Co., Ltd., Qingdao, China) and incubated at 28 °C for 7–15 days, respectively. The colonies were separated according to the morphological characteristics such as colony color, growth time, and size. Each colony was purified on yeast extract–malt extract (ISP2) agar (Qingdao Hope Bio-Technology Co., Ltd., Qingdao, China) and kept in 20% (*v*/*v*) of glycerol at −80 °C. Foc TR4 was stored in the Institute of Tropical Bioscience and Biotechnology, Chinese Academy of Tropical Agricultural Sciences.

### 2.2. Screening of Antifungal Actinomycetes

A conventional spot inoculation method was used to determine the inhibition ability of the purified strains against Foc TR4 [21]. A phytopathogenic fungal disk (8.5 mm diameter) was placed in the center of the potato dextrose agar (PDA) plate. Four mycelial blocks (5 mm diameter) of each actinomycete were inoculated at four symmetrical points about 2.5 cm from the plate center. A fungal piece of Foc TR4 alone was used as a control. An agar well-diffusion method was performed to test the antifungal activity of ethanol extracts on the potato dextrose agar (PDA) medium. Four holes were punched in four vertical positions and 50 μL of extracts were added to measure the antifungal activity of the fermentation broth. An equal volume of water was used to replace extracts as a control. After incubation at 28 °C for 5–7 days, the growth diameters of Foc TR4 were measured by a cross method [22]. The inhibition activity was measured by the inhibition rate. Inhibition rate (%) = [(A−B)/(A−0.5)] × 100, where A and B were the mean colony diameters (cm) of phytopathogenic fungi in the control and treatment groups, respectively. All strains were tested in triplicate experiments.

### 2.3. Identification of Antifungal Actinomycetes

The screened isolates were cultured in ISP2, ISP3, ISP4, ISP5, ISP6, ISP7 (Qingdao Hope Bio-Technology Co., Ltd., Qingdao, China) and PDA media at 28 °C for 7–10 days. The morphological features including mycelia and spores as well as the color of aerial mycelia were measured [18]. We also carried out physiological and biochemical identification of the isolate, such as pH and NaCl tolerance, soluble pigment, H_2_S production, urease and carbon and nitrogen utilization [23]. In addition, the screened isolates were identified by molecular biology [24]. The total DNA of the selected isolate was extracted using a Rapid Bacterial Genomic DNA Isolation Kit (Aidlab biotechnologies Co., Ltd., Beijing, China). The 16S rRNA sequence was amplified using a pair of universal primers (27F: 5′-AGAGTTTGA TCCTGGCTCAG-3′ and 1492R: 5′-TACCTTGTTACGACTT-3′). PCR was performed in a Biometra Professional Gradient Thermocycler (Biometra-T Gradient, Whatman Biometra, Göttingen, Germany). The PCR reaction system included denaturation at 94 °C for 5 min, followed by 32 cycles (94 °C for 10 s, 57 °C for 30 s, and 72 °C for 1 min 30 s), and a final extension at 72 °C for 5 min. PCR products were detected by 1% (*w*/*v*) of agarose gel electrophoresis and sequenced by the Nanshan Biotech Co., Ltd. (Haikou, China). The sequence was aligned against the database of NCBI (https://www.ncbi.nim.nih.gov, accessed on 12 August 2021). The multiple sequence alignment was carried out using the Clustal W software (Arizona State University, Tempe, AZ, USA). The phylogenetic tree was constructed using the Neighbor-Joining (NJ) method of MEGA software (Version 7.0, Mega Limited, Auckland, New Zealand) [25].

### 2.4. Assay of Abroad-Spectrum Antifungal Activity of Strain Y1-14

To test the broad-spectrum antifungal activity of strain Y1-14 and its extracts, seven fungal phytopathogens including *Colletotrichum gloeosporioides*, *Alternaria tenuissima*, *Curvularia fallax*, *Colletotrichum gloeosporioides Penz*, *Curvularia lunata*, *Pyricularia oryae Cav*, *Cryphonectria parasitica (Murr.) Barr*, *Ustilaginoidea virens*, *Fusarium graminearum Schwabe*, *Fusarium oxysporum* f. sp. *cucumerinum*, *Fusarium oxysporum* f. sp. *cubense race 1*, and *Colletotrichum acutatum* were provided by the Key Laboratory of Biology and Genetic Resources of Tropical Crops, Ministry of Agriculture, Institute of Tropical Bioscience and Biotechnology, Chinese Academy of Tropical Agricultural Sciences, Haikou, China. Antifungal activity was measured by a conventional spot inoculation method. All experiments were repeated in triplicate.

### 2.5. Preparation of Ethanol Extracts

Strain Y1-14 was inoculated in one liter of sterilized soybean liquid culture medium (SLM, 15 g of corn flour, 10 g of glucose, 0.5 g of K_2_HPO_4_, 0.5 g of NaCl, 0.5 g of MgSO_4_, 3 g of beef extracts, 10 g of yeast extracts, 10 g of soluble starch, and 2 g of CaCO_3_; pH 7.2–7.4) at 180 rpm for 7 d at 28 °C. Then, culture filtrate was extracted with 95% of ethanol at a ratio of 1:1 (*v*/*v*). The mixture was filtered through the qualitative filter paper (Whatman no. 1). The extracts were evaporated using a rotary vacuum evaporator (EYELA, N-1300, Tokyo, Japan). Crude extracts were eluted using a linear gradient of methanol: deionized water (100:0) on a silica gel column (8.0 inner diameter, 60 cm length). The extracts were dissolved in 10% of dimethyl sulfoxide (DMSO) with a final concentration of 20.0 mg/mL. The extracts were sterilized by a 0.22 µm sterile filter (Millipore, Bedford, MA, USA) and stored at −4 °C [26].

### 2.6. Inhibition Percentage of Strain Y1-14 Extracts on Mycelial Growth

The effect of strain Y1-14 extracts on mycelial growth of Foc TR4 was investigated. Extracts were added to the sterilized PDA medium and diluted into different concentrations of 0.78 mg L^−1^, 1.56 mg L^−1^, 3.12 mg L^−1^, 6.25 mg L^−1^, 12.50 mg L^−1^, 25.00 mg L^−1^, and 50.00 mg L^−1^, respectively [27]. The same concentration of DMSO was used as a negative control. A 5 mm diameter fungal disc was placed on the center of the plate. The growth diameter of Foc TR4 was recorded after 7 d at 28 °C. All experiments were performed in triplicate. A half maximal effective concentration (EC_50_) value was calculated according to the toxicity regression equation [28].

### 2.7. Effect of Strain Y1-14 Extracts on Morphological Characteristics of Foc TR4 Mycelia

The mycelial characteristics of Foc TR4 treated with extracts of strain Y1-14 were observed by a scanning electron microscope (SEM, TM4000Plus, Hitachi, Tokyo, Japan). A 5 mm Foc TR4 disc was inoculated on a PDA plate containing 1 × EC_50_ of extracts. The samples were cut from the mycelial edge with a 1 cm^2^ cross section and a 3 mm thickness. The same volume of DMSO was used as a control. The samples were fixed in 2.5% (*v*/*v*) of glutaraldehyde for 4 h and rinsed four times with PBS for 20 min each time. Dehydration was carried out with alcohol solutions of different concentrations (30%, 50%, 70%, 90%, and 100%). The dehydrated samples were immersed in a solution of tert-butyl alcohol for 15 min and dried using carbon dioxide. The dried samples were coated with thin gold and observed using SEM.

A 5 mm Foc TR4 disc was inoculated on the PDA plate. The sterile glass slides were placed in the plate at a distance of 5 mm from the Foc TR4 disc. After being cultured at 28 °C for 3 d, 500 µL of strain Y1-14 extracts (8 × EC_50_) were dropped onto a slide for 1 d. The mycelial growth was detected by a fluorescence microscope (MMI Cell Cut Plus, Leica DM6000B, Wetzlar, Germany).

### 2.8. Effect of Strain Y1-14 Extracts on Spore Germination of Foc TR4

The spore germination of Foc TR4 was observed by a light microscope (Nikon, E200MV, Tokyo, Japan) after treatment with strain Y1-14 extracts. The spore suspension (1.0 × 10^6^ CFU/mL) of Foc TR4 was fully mixed with the different concentrations of extracts (1 × EC_50_, 2 × EC_50_, 4 × EC_50_, and 8 × EC_50_). The mixture was dropped onto sterile glass slides and incubated at 28 °C for 12 h. The sterile water was used as a negative control. Spore germination was evaluated using the inhibition efficiency [29]. All experiments were performed in three biological replicates.

### 2.9. Effects of Strain Y1-14 Extracts on Ultrastructure of Foc TR4

Sample collection, fixation, and dehydration were described as the above methods. After that, the treated samples were embedded into Epon 812 resin at 37 °C for 12 h, 45 °C for 12 h, and 60 °C for 24 h, respectively. The embedded materials were sectioned with an ultramicrotome (EM UC6, Leica, Wetzlar, Germany) and stained with uranyl acetate and lead citrate solution. The ultrastructure of mycelia was observed by a transmission electron microscope (TEM, HT7700, Hitachi, Ibaraki, Japan).

### 2.10. Determination of Antifungal Activity of Volatile Organic Compounds from Strain Y1-14

Double plate assay was used to study the role of volatile organic compounds (VOCs) in inhibiting Foc TR4 [30]. A 5 mm strain Y1-14 disc was inoculated on the PDA plate and incubated at 28 °C for 5 days. The same size of Foc TR4 disc was inoculated into other PDA plates. A petri plate containing the pathogenic fungus was inverted over the petri plate inoculated with Y1-14. Mycelia disc of pathogen or Y1-14 alone was used as a control. After incubation at 28 °C for 7 days, the inhibition percentage was calculated as described earlier. All experiments were repeated in triplicate [31].

### 2.11. Component Analysis of VOCs by Head-Space Gas Chromatography–Mass Spectrometry

To obtain the VOCs of strain Y1-14, the strain was incubated in PDB medium in a conical flask. After incubation for 7 d at 28 °C, VOCs were collected and determined using a head-space solid phase micro-extraction (SPME, 57324-a) coupled with gas chromatography–mass spectrometry (GC-MS). The SPME syringe was used to trap VOCs. An SPME fiber coated with DVB/CAR/PDMS was exposed to the tapered bottles to absorb VOCs emitted by strain Y1-14 for 30 min at 50 °C. The treated fiber was then immediately put into the GC inlet to analyze the components of the VOCs. The desorbed VOCs were injected into the GC port in a split less mode at 250 °C and separated in a PerkinElmer SQ8T gas chromatograph coupled with PerkinElmer TGA8000 mass spectrometer equipped with a GC column Elite-5 MS column (30 m × 0.25 mm × 0.25 µm.). The oven temperature was set at 40 °C for 1 min hold time and then increased from 40 °C to 160 °C at the rate of 3 °C/min, from 160 °C to 230 °C at the rate of 15 °C/min for 2 min, and from 230 °C to 250 °C at a rate of 10 °C/min for 5 min hold time. The transfer line was heated at 250 °C [32]. The flow rate of the helium carrier gas was 1 mL/min. The components of VOCs were identified by matching the mass spectral data of the peak with library spectra (NIST Library). Head-space samples taken from sterile PDA served as negative control in order to exclude interfering substances.

### 2.12. Effects of Strain Y1-14 Extracts on Metabolism of Foc TR4

Foc TR4 was inoculated on the PDB liquid medium and cultured for 3 d. Strain Y1-14 extracts were dissolved in 10% (*v*/*v*) of DMSO with the final concentration 1 × EC_50_. The same concentration of DMSO was used as a negative control. The samples were collected after treatment at 0 h, 6 h, and 12 h. The spore solution was filtered through two layers of filter paper to collect the treated mycelia. The materials were sent to the Biomarker Technology Company (Beijing, China) for metabolomics analysis.

The LC/MS system for metabolomics analysis was composed of Waters Acquity I-Class PLUS ultra-high performance liquid tandem Waters Xevo G2-XS QT high-resolution mass spectrometer. Acquity UPLC HSS T3 column (1.8 µm × 2.1 mm × 100 mm) was purchased from Waters (Waters Corporation, Milford, MA, USA). Waters Xevo G2-XS QTOF high-resolution mass spectrometer was used to collect primary and secondary mass spectrometry data in MSe mode (MassLynx V4.2, Waters). MassLynx V4.2 was used to collect the raw data, and Progenesis QI software (Waters, Wilmslow, UK) was used for peak extraction, peak alignment, and other data processing operations. Progenesis QI software online METLIN database and Biomark’s self-built library were used for identification and theoretical fragment identification. Quality deviation fell within 100 ppm.

### 2.13. Determination of Mitochondrial Enzyme Activities

After Foc TR4 mycelia were treated with Y1-14 extracts, mycelial mitochondria were isolated by sucrose differential centrifugation [33]. The mycelia were washed three times with 5 mL of pre-cooled HEPES-Tris buffer (20 mM, pH 7.2) and resuspended in pre-cooled mitochondrial extraction buffer (250 mM sucrose, 10 mM KCl, 5 mM EDTA, 20 mM HEPES-Tris, pH 7.2, 1.5 mg/mL BSA). The mycelial samples were homogenized with a glass bead vortex oscillator and centrifugated at 4 °C and 4000 rpm for 15 min. The collected supernatant was centrifugated at 12,000 rpm at 4 °C for 10 min. The precipitate was washed with mitochondrial extraction buffer without BSA and kept as the crude extract of mitochondrial granules. The extracted mitochondrial particles were used as materials to determine succinate dehydrogenase (SDH), malate dehydrogenase (MDH), and ATP synthase according to the commercial kit (Solarbio Life Science Co., Ltd., Beijing, China). All experiments were repeated in triplicate.

### 2.14. Data Analysis

Statistical analysis was performed with the SPSS 23 Version software (SPSS Inc., Chicago, IL, USA). Significant difference between means was determined by the Duncan’s multiple range test at *p* < 0.05. The difference among treatments was determined using one-way analysis of variance (ANOVA). All data were expressed as means ± the standard error (SE) from three biological replicates of each experiment.

## 3. Results

### 3.1. Screening and Identification of Actinomycetes with Antifungal Activity

**Figure 1:**
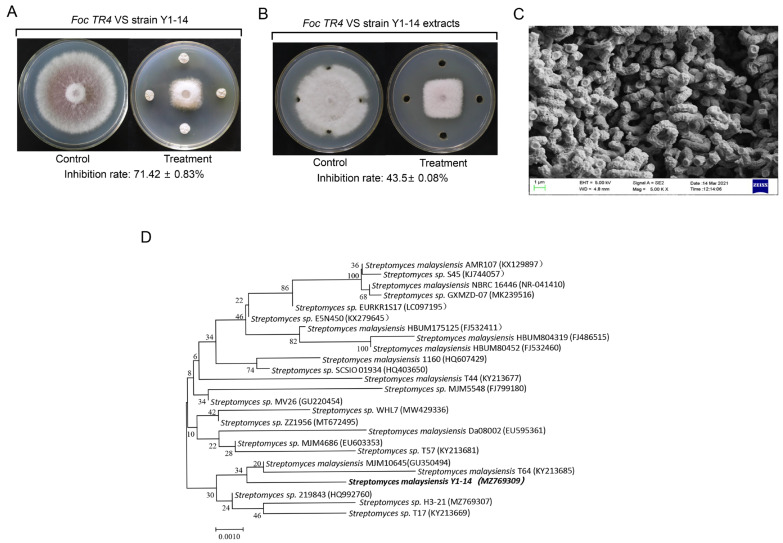
Isolation and identification of strain Y1-14 with strong antifungal activity against Foc TR4. (**A**) Strain Y1-14 inhibiting mycelial growth of Foc TR4. (**B**) Strain Y1-14 extracts inhibiting mycelial growth of Foc TR4. (**C**) Morphological characteristics of aerial mycelia and spores of strain Y1-14 using SEM. (**D**) Phylogenetic tree of strain Y1-14 based on 16S rRNA gene sequence analysis. The bootstrap values (%) at the branches were calculated from 1000 replications.

Seventeen actinomycetes with antagonistic activity against Foc TR4 were isolated from the non-pathogenic banana plantation soils. Especially, an isolate labeled with Y1-14 had the strongest antifungal activity against Foc TR4 and exhibited 71.42% of growth inhibition rate (Figure 1A). The extracts of strain Y1-14 can also inhibit the mycelial growth of Foc TR4 with 43.5% of the inhibition rate (Figure 1B).

Strain Y1-14 produced burlywood pigment on ISP4 medium and pink soluble pigment on ISP6 medium. Pigment was not detected on other selected medium (Supplementary Table S1). It can grow normally under the conditions of pH ranging from 4 to 10 and less than 5% of NaCl. Strain Y1-14 could produce H_2_S, hydrolyze starch, solidify milk, decompose cellulose, but could not hydrolyze nitrate and gelatin liquefaction (Supplementary Table S2). It could utilize ten selected nitrogen sources and ten selected carbon sources (Supplementary Table S3). SEM was used to detect the morphological characteristics of strain Y1-14. The open spirals of spore-bearing mycelia were observed. (Figure 1C). According to the above morphological, physiological, and biochemical characteristics, strain Y1-14 was preliminarily identified as *Streptomyces* sp.

To further verify the relationship between strain Y1-14 and *Streptomyces* sp., the 16S rRNA sequence was amplified by PCR and deposited in GenBank with an accession number MZ769309. In comparison with the database of EzBioCloud, the 16S rRNA sequence of strain Y1-14 showed 99% of similarity with *Streptomyces malayensis*. The phylogenetic tree was constructed by the Neighbor-Joining (NJ) method. The results showed that Y1-14, GU350494, and KY213685 were clustered into the same branch (Figure 1D). Combining with the morphological, physiological, and biochemical characteristics as well as the alignment result of 16S rRNA, strain Y1-14 was identified as the genus of *Streptomyces*.

### 3.2. Assay of Broad-Spectrum Antifungal Activity

**Figure 2:**
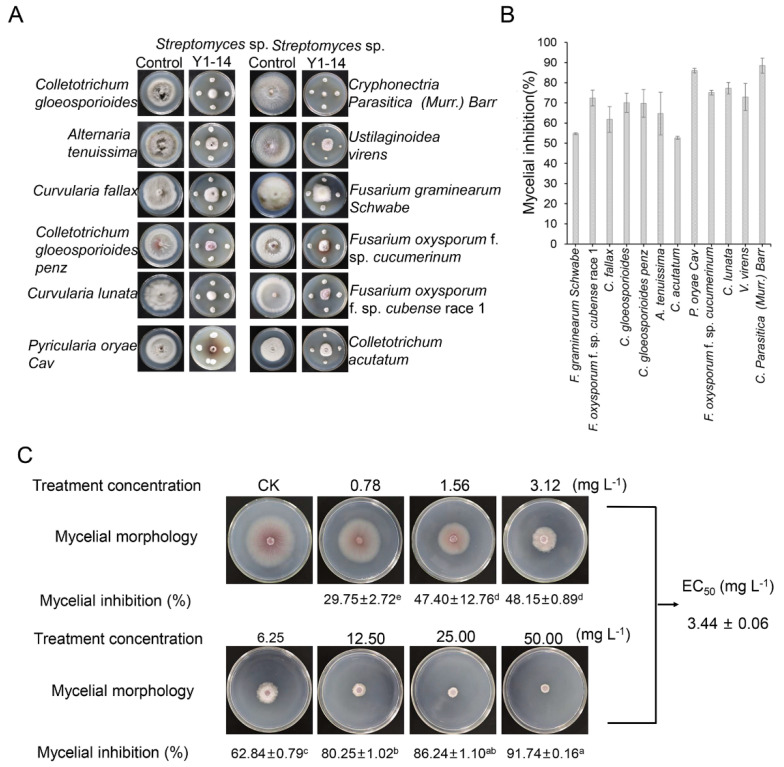
(**A**) A broad-spectrum antifungal activity of *Streptomyces* sp. Y1-14 against the selected twelve phytopathogenic fungi. (**B**) The mycelial inhibition (%) of Foc TR4 after treatment with *Streptomyces* sp. Y1-14. (**C**) Effects of *Streptomyces* sp. Y1-14 extracts on mycelial growth of Foc TR4. Different lowercase letters indicated a significant difference according to the Duncan’s multiple range test (*p* < 0.05).

The broad-spectrum antifungal activities of *Streptomyces* sp. Y1-14 were examined against 12 fungal phytopathogens by the conventional spot inoculation method. The results showed that *Streptomyces* sp. Y1-14 inhibited the growth of fungal phytopathogens. The inhibition rate ranged from 52.6% to 88.5%. *Streptomyces* sp. Y1-14 showed maximal inhibition activity against *C. parasitica* (*Murr*.) *Barr* and minimal inhibition activity against *C. acutatum* (Figure 2A,B).

### 3.3. Inhibition Activity of Streptomyces sp. Y1-14 against Foc TR4

Increasing evidence showed that secondary metabolites of *Streptomyces* had a capacity of inhibiting the growth of fungal pathogens. To verify whether the metabolites of *Streptomyces* sp. Y1-14 had antagonistic activity against Foc TR4, the crude extracts of strain Y1-14 were extracted with different concentrations of methanol (50–100%, *v*/*v*). The antagonistic activity of extracts increased significantly along with the increase in methanol concentrations. Antagonistic activity of extracts was strongest in 100% methanol. Hence, extracts of *Streptomyces* sp. Y1-14 isolated with 100% of methanol were selected for further study. The antagonistic activity was measured against Foc TR4 after culture for 7 days. Compared with the control group, the inhibitory rate was 29.75 ± 2.72 after treatment with 0.78 mg/L extracts. When extract concentration was 50 mg/L, the inhibitory rate was 91.74 ± 0.16 (Figure 2C). By calculation, the EC_50_ value against Foc TR4 was 3.44 ± 0.06 mg /L, which was defined as 1 × EC_50_ in a follow-up study.

### 3.4. Effect of Streptomyces sp. Y1-14 Extracts on Spore Germination and Germ Tube Elongation of Foc TR4 In Vitro

**Figure 3:**
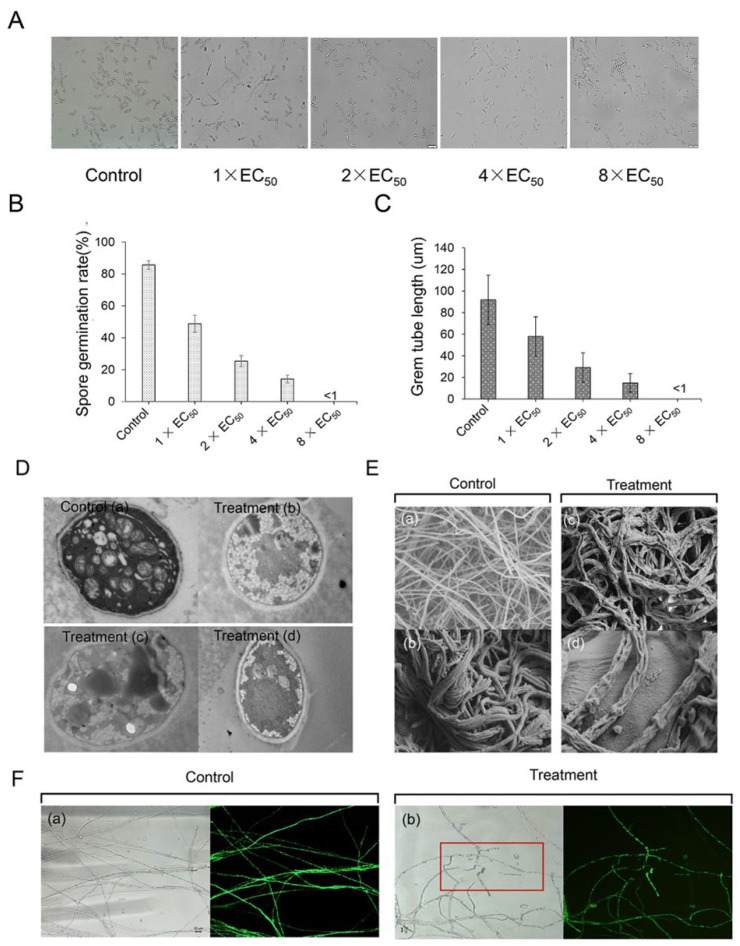
Inhibition of *Streptomyces* sp. Y1-14 extracts on spore germination of Foc TR4. (**A**) Spore germination characteristics of Foc TR4 after treatment with 1 ×, 2 ×, 4 ×, or 8 × EC_50_ extracts. A 10% concentration of DMSO was used as a control. Bar = 20 µm. (**B**) The spore germination rate (%) of Foc TR4 after treatment with different dose extracts. (**C**) The germ tube length (µm) of Foc TR4 after treatment with different dose extracts. (**D**) Mycelial cell ultrastructure of Foc TR4 observed under TEM. (**a**) Ultrastructure characteristics of Foc TR4. (**b**–**d**) Ultrastructure characteristics of Foc TR4 treated with 4 × EC_50_ of *Streptomyces* sp. Y1-14 extracts. Bar = 2 µm. (**E**) Mycelial morphology of Foc TR4 observed under SEM. (**a**,**b**) Mycelial characteristics of Foc TR4 in the absence of stain Y1-14 extracts. (**c**,**d**) Mycelial morphology of Foc TR4 treated with 4 × EC_50_ of *Streptomyces* sp. Y1-14 extracts. Bar = 10 µm (**F**) Mycelial morphology of Foc TR4 observed under fluorescence microscope. (**a**) Mycelial characteristics of Foc TR4 in the absence of *Streptomyces* sp. Y1-14 extracts. (**b**) Mycelial morphology of Foc TR4 treated with 8 × EC_50_ of *Streptomyces* sp. Y1-14 extracts. The GFP gene disap-peared site was marked with the red frame. Bar = 20 µm.

*Streptomyces* sp. Y1-14 extracts also effectively inhibited the spore germination and germ tube elongation of Foc TR4. Compared with the control group, the spore germination rates were 48.76%, 25.28%, and 14.17% after treatment with 1 × EC_50_, 2 × EC_50_, and 4 × EC_50_ for 12 h, respectively (Figure 3A). The inhibitory efficiency was enhanced along with the increase in the concentration of the extracts. Especially, when the concentration of the extract was 8 × EC_50_, the spores could rarely germinate and the germination rate was less than 1% (Figure 3B). Similarly, germ tube lengths of Foc TR4 were significantly inhibited. In comparison with 91.87 μm in the control group, germ tube lengths were 57.87 μm, 28.87 μm, and 14.87 μm after being treated with 1 × EC_50_, 2 × EC_50_, and 4 × EC_50_ extracts, respectively. The length of the germ tube significantly failed to develop at the treatment group of 8 × EC_50_ extracts (Figure 3C).

### 3.5. Effect of Streptomyces sp. Y1-14 Extracts on Mycelial Morphology and Ultrastructure of Foc TR4

The disruption of Foc TR4 hyphae by extracts of *Streptomyces* sp. Y1-14 was evaluated by microscopic and submicroscopic structure. In the control group, the surface of Foc TR4 hyphae was smooth, the thickness of the mycelial was uniform and the distribution of the mycelial was regular. After being treated with 4 × EC_50_ of *Streptomyces* sp. Y1-14 extracts, the hyphae showed shrinkage morphology, suggesting that the extracts destroyed the mycelial structure of Foc TR4 (Figure 3E). Similarly, the fluorescence signals of GFP-Foc TR4 overexpressing the GFP gene disappeared. The cytoplasmic membrane became dissolved after treatment with 8 × EC_50_ of extracts (Figure 3F). We further evaluated the effects of *Streptomyces* sp. Y1-14 extracts on ultrastructural changes of Foc TR4 cells by TEM. In the control cells, intact cell membranes and cell walls were observed clearly. Some complete organelles such as vacuoles, cell nuclei, and mitochondria were also demonstrated. After being treated with 4 × EC_50_ of *Streptomyces* sp. Y1-14 extracts, Foc TR4 cells showed organelle dissolution, structural disorder, and blurred matrix. Cell membrane integrity was also broken and disintegrated gradually (Figure 3D).

### 3.6. Chemical Constituent Analysis of VOCs

**Figure 4:**
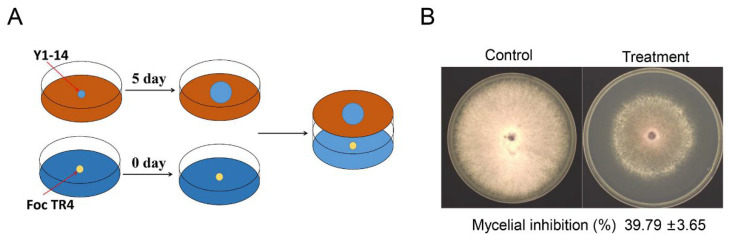
Antifungal activity of VOCs produced by *Streptomyces* sp. Y1-14 against Foc TR4. (**A**) Double plate assay was used to evaluate the antagonistic activity of *Streptomyces* sp. Y1-14. The blue circle represented the disc of *Streptomyces* sp. Y1-14 and the yellow circle represented the disc of Foc TR4. (**B**) VOCs produced by *Streptomyces* sp. Y1-14 inhibiting mycelial growth of Foc TR4.

To detect antifungal activities of VOCs isolated from *Streptomyces* sp. Y1-14, the double plate assay was carried out (Figure 4A). After 7 days of co-culture, the mycelial growth of Foc TR4 was significantly inhibited by *Streptomyces* sp. Y1-14, and the inhibition rate was 39.79% ± 3.65 (Figure 4B). The antifungal components of VOCs produced by *Streptomyces* sp. Y1-14 were analyzed by GC-MS. Based on the total integrated peak areas, the emission spectrum showed that the extracts of *Streptomyces* sp. Y1-14 contained 22.75% of terpene, 22.46% of alcohols, 25.96% of hydrocarbons, and 28.81% of other components (Table 1). Among these high levels of substances, trans-1,10-Dimethyl-trans-9-decalinol, 2-Methylisoborneol, β-Cubebene, β-Chamigrene, and nerolidol possessed antifungal, antimicrobial, insecticidal, and antitumor activities [34,35,36,37,38]. By experiment validation, the effects of these substances on the growth of Foc TR4 were measured. Compared with the control group, the inhibition rates of nerolidol at 800 μM, 400 μM, 200 μM, 100 μM, 50 μM, and 0 μM were 67.75% ± 3.65, 53.72% ± 6.72, 28.87% ± 2.84, 26.97% ± 4.57, and 17.02% ± 1.64, respectively (Supplementary Figure S1A,B).

### 3.7. Effects of Streptomyces sp. Y1-14 Extracts on Foc TR4 Metabolites

**Figure 5:**
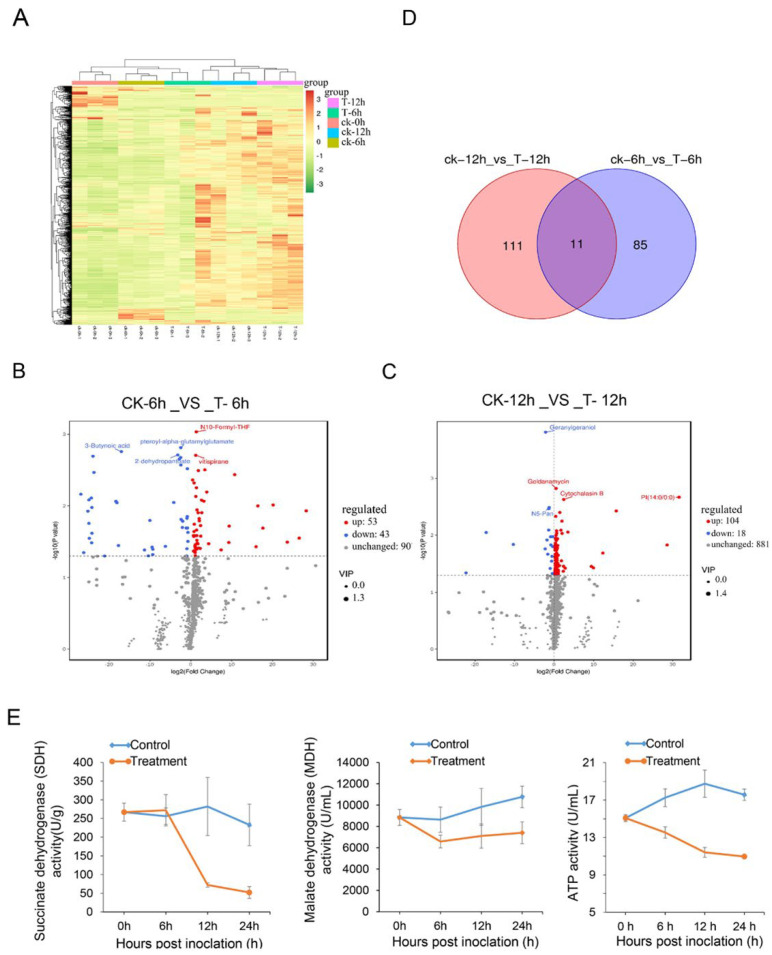
Effect of *Streptomyces* sp. Y1-14 on secondary metabolism of Foc TR4. (**A**) Heatmap based on the metabolome data of Foc TR4 treated with extracts of *Streptomyces* sp. Y1-14 at 0 h, 6 h, and 12 h. Three biological replications were carried out for analyzing the metabolite profiling. (**B**,**C**) Volcanic map of differential metabolites. Each dot represents a metabolite, the abscissa represents the group contrast ratio of each material change (with 2 logs base), the vertical ordinate represents the *p*-*t* test value (in the logarithm of 10 at the bottom), and the size of scatter represents the VIP value of the OPLS-DA model, the larger the scatter, the greater the VIP value; the differential expressed metabolites obtained by screening were more reliable. Blue dots in the figure represent downregulated DEMs, red dots represent upregulated DEMs, and grey dots represent no significantly different metabolites. In addition, the top 5 qualitative metabolites were selected and labeled in the figure after sorting by *p* value. (**D**) Venn diagram of the metabolite numbers. Ck-12 h vs. T-12 h represents DEMs of Foc TR4 treated with *Streptomyces* sp. Y1-14 extracts compared with the control at 12 h. Ck-6 h vs. T-6 h represents DEMs of Foc TR4 treated with *Streptomyces* sp. Y1-14 extracts compared with the control at 6 h. (**E**) Determination of enzyme activities of Foc TR4 after treatment with *Streptomyces* sp. Y1-14 extracts.

To further analyze the secondary metabolites of *Streptomyces* sp. Y1-14 extracts-treated Foc TR4, the analysis of widely targeted metabolome was performed using the LC-ESI-MS/MS system. A total of 1003 metabolites were obtained in control and treatment samples (Figure 5A). The principal component analysis (PCA) revealed that PC1, PC2, and PC3 were 68.73%, 25.74%, and 3.98% in 3D diagram, respectively (Supplementary Figure S2A). Compared with the metabolites of Foc TR4 in the control group, the numbers of upregulated and downregulated metabolites after 6 h of treatment were 53 and 43, respectively (Supplementary Figure S2B). N10-Formyl-THF and vitispirane were significantly upregulated. 3-Butynoic acid, 2-dehydropantoateand, and pteroyl-alpha-glutamyl glutamate were significantly downregulated (Figure 5B). According to functional annotation, N10-Formyl-THF and 2-dehydropantoate belonged to the carbon metabolism pathway (ko01200) as well as pantothenate and CoA biosynthesis (ko01110), respectively. In the growth tested after treatment for 12 h, 122 metabolites showed differences, 104 metabolites were upregulated, and 18 metabolites were downregulated (Supplementary Figure S2C). Especially, cytochalasin B and geldanamycin were significantly upregulated. N5-Pan and geranylgeraniol were obviously downregulated (Figure 5C). A Total of 207 differentially expressed metabolites (DEMs) were majorly classified into organooxygen compounds, glycerophospholipids, carboxylic acids and derivatives, fatty acyls, prenol lipids, and imidazopyrimidines, etc (Supplementary Figure S2D,E). The Venn diagram was used to further analyze the intersection and uniqueness of metabolites in different differential groups. Eleven differential metabolites were shared by two differential groups (Figure 5D). The results of KEGG annotation showed that eleven metabolites were clustered into pentose phosphate pathways and carbon metabolism pathways, suggesting that *Streptomyces* sp. Y1-14 extracts affected the energy metabolism of Foc TR4.

We further detected the activities of key enzymes in TCA cycle after Foc TR4 hyphae was treated with *Streptomyces* sp. Y1-14 extract. Activity of ATP, MDH, and SHD in hyphae were detected after treatment with 1 × EC_50_ of *Streptomyces* sp. Y1-14 extracts for 6 h, 12 h, and 24 h. Compared with the control group, ATP and MDH activities of hyphae were reduced in 6 h, 12 h, and 24 h. SDH activity did not differ significantly after treatment for 6 h, but was reduced for 12 h and 24 h (Figure 5E). These results suggested that *Streptomyces* sp. Y1-14 extracts affected energy metabolism and inhibited the growth of Foc TR4.

## 4. Discussion

In comparison with traditional pesticide methods, biocontrol has a low investment cost and eco-friendly characteristics [29]. Actinomycetes are one of the most important resources for biocontrol. Most *Streptomyces* have broad-spectrum antifungal activities in controlling plant diseases [21]. In our previous studies, *Streptomyces* sp. WHL7 was isolated from a marine soft coral and significantly inhibited the growth of Foc TR4 [18]. However, the ecological environment of a banana plantation is different from a marine environment, making it less effective to use in banana plantation. In our present study, *Streptomyces* sp. Y1-14 was isolated from the non-Foc TR4 disease soil in the banana plantation and exhibited a strong antifungal ability against Foc TR4. Considering *Streptomyces* sp. Y1-14 isolated from banana plantations, it is more conducive to application in the field. Interestingly, the strain also exhibited antifungal ability against different phytopathogenic fungi, suggesting that *Streptomyces* sp. Y1-14 has great potential in managing plant diseases caused by fungal pathogens.

Microbial VOCs play an important role in microbial communities [39,40]. *Streptomyces* are known to emit VOCs with various antifungal components [41]. In this study, the antifungal ability of Y1-14 VOCs against Foc TR4 was detected by double plate analysis and it was found that it could effectively inhibit the growth of Foc TR4. Upon further investigation of the antifungal mechanism of strain Y1-14 VOCs against Foc TR4, it was found that the main components were terpenes, alcohols, and hydrocarbons. The highest contents of trans-1,10-Dimethyl-trans-9-decalinol and 2-Methylisoborneol were in VOCs. The trans-1,10-Dimethyl-trans-9-decalinol in *Streptomyces alboflavus* TD-1 was the major antifungal, and was proven to be responsible for the inhibitory activity against *F. moniliforme* [34]. 2-Methylisoborneol (2-MIB) in actinomycetes affected sea urchin development and the germination of some Brassicaceae seeds [35,36]. The antifungal properties of 2-MIB were reported against *F. moniliforme* as fumigants [34]. These indicated that VOCs produced by actinomycetes were rich in antifungal substances to inhibit the growth of phytopathogenic fungi.

In addition to VOCs, extracts of *Streptomyces* can also inhibit the growth of pathogenic fungi. In our present study, the inhibition rate of Foc TR4 mycelial growth reached 91.74% under the treatment of 50 mg/L extracts. The treated hyphae became wrinkled and the cytoplasmic membrane was disrupted. Foc TR4 spore gemination was also significantly inhibited after being treated with 8 × EC_50_ of extracts. Previous studies indicated that natural products produced by *Streptomyces* species were used as antibiotics, antitumor agents, antioxidants, pesticides, and plant-growth-promoting substances, etc. [24,42]. About 2/3 of clinically useful antibiotics were produced by *Streptomyces* sp., including many important drugs, such as streptomycin, neomycin, kanamycin, rapamycin, chloramphenicol, vancomycin, etc. [43]. Therefore, the extracts of *Streptomyces* sp. YI-14 were expected to be used in other fields for inhibiting the growth of pathogenic fungi.

Our previous study indicated that *Streptomyces* sp. JBS5-6 extracts could break and disintegrate Foc TR4 mycelia [26]. *Streptomyces* sp. H3-2 suppressed the growth and spore germination of Foc TR4 in vitro by destroying cell membrane integrity and mycelial ultrastructure [22]. However, the antibacterial mechanism is not clear. To further investigate that *Streptomyces* sp. Y1-14 extracts affected the production of Foc TR4 metabolites, metabolomics was used to identify the critical metabolism pathways. By contrast, high abundance of metabolites mainly clustered into the carbon metabolism pathway (central carbohydrate metabolism, CCM) and pentose phosphate pathway. Carbohydrate pathways played an essential role in energy generation and biomass formation in organisms of all life domains. The central carbon metabolism of heterotrophs plays an essential role in the conversion of biomass to cellular building [44]. The CCM in microorganisms provided energy in the form of ATP [45]. Glycolysis, is generally considered to be the breakdown of glucose to pyruvate in the CCM of most prokaryotes especially heterotrophs [46]. Glycolytic pathways can be classified into five categories: Embden–Meyerhof–Parnas (EMP)-derived pathways, Entner–Doudoroff (ED)-derived pathways, pentose phosphate (hexose monophosphate) pathways, methylglyoxal pathways, and phosphoketolase pathways [47].

The pentose phosphate pathway (PPP) is a route that can work in parallel to glycolysis in glucose degradation in most living cells [48]. Apart from glycolysis, the pentose phosphate pathway is a major route of intermediary carbohydrate metabolism in enteric bacteria and fulfills various roles in these organisms [49]. In addition to the breakdown of carbon sources such as glucose or gluconate, the pentose phosphate pathway is involved in the generation of reducing power (NADPH) for biosynthesis and the recruitment of essential metabolites for nucleic acids, amino acids, and vitamins [50]. This suggests that the carbohydrate metabolism of Foc TR4 was significantly affected.

To verify the effect of *Streptomyces* sp. Y1-14 extracts on Foc TR4 energy metabolism, activities of ATP, MDH, and SDH in the tricarboxylic acid (TCA) cycle were significantly inhibited. The respiratory pathways of glycolysis, the TCA cycle, and the mitochondrial electron transport chain are ubiquitous throughout nature [51]. They are essential for both energy provision in heterotrophic cells and a wide range of other physiological functions [52]. TCA has been known for decades as a hub for generating cellular energy and precursors for biosynthetic pathways [53]. The enzyme that catalyzed the dehydrogenation of succinic acid to fumaric acid is called SDH. SDH catalyzed the interconversion of a range of related sugar alcohols into their corresponding ketoses [54]. It is one of the marker enzymes reflecting mitochondrial function, and its activity can generally be used as an indicator to evaluate the degree of tricarboxylic acid cycle operation. SDH has been identified as one of the most significant targets for fungicide discovery [55]. MDH is a widely distributed enzyme, and plays key roles in many important pathways [56]. MDH, with the help of a cofactor nicotinamide adenine dinucleotide (NAD^+^/NADH), catalyzed oxidation/reduction of malate/oxaloacetate, an important step in glycolysis. In our study, *Streptomyces* sp. Y1-14 extracts disrupted Foc TR4 cell structure, leading to mitochondrial dissolution. Therefore, *Streptomyces* sp. Y1-14 extracts affected mitochondrial structure of Foc TR4, resulting in the inhibition of energy metabolism.

## 5. Conclusions

*Streptomyces* sp. Y1-14 with a strong antifungal activity against Foc TR4 was isolated from the rhizosphere soil of banana. Its extracts effectively inhibited the mycelial growth and spore germination of Foc TR4. *Streptomyces* sp. Y1-14 also showed a broad-spectrum antifungal activity against 12 pathogenic fungi. Twenty-three compounds were identified by GC-MS, and nerolidol was tested for antifungal activity. After treatment with *Streptomyces* sp. Y1-14 extracts, the ultrastructure of Foc TR4 cells exhibited a deformation feature and the organelles gradually disappeared. The energy metabolism pathway of Foc TR4 was seriously disrupted.

## Figures and Tables

**Table 1:** VOCs produced by *Streptomyces* sp. Y1-14 by GC-MS analysis.

RT ^a^ (min)	Possible Compound ^b,c^	Relative Peak Area (%)	Classification
1.343	Ala-Gly	1.4163	Others
2.081	Ethyl Acetate	1.0644	
4.089	Silanediol, dimethyl-	1.0444	
11.026	3-Furancarboxylic acid, methyl ester	1.1225	
30.66	trans-1,10-Dimethyl-trans-9-decalinol	21.05	
12.949	*p*-Menth-8-ene, 3-methylene-	3.9783 f	Hydrocarbons
22.687	Naphthalene, 1,2,3,4,4a,5,6,8a-octahydro-4a,8-dimethyl-	9.5415	
22.894	Cyclohexane, 1,1,4,4-tetramethyl-2,5-dimethylene-	4.0992	
23.551	Bicyclo [4.3.0] non-3-ene, 3,4,7-trimethyl-	1.1359	
23.74	(2Z)-2-ethylidene-1,7,7-trimethylbicyclo [2.2.1] heptane	2.2966	
30.34	Tetradecane	1.1357	
20.836	2-Methylisoborneol	11.9192	Alcohols
32.681	Octahydro-2,2,5a,9-tetramethyl-2H-3,9a-methano-1-benzoxepin	3.8177	
35.365	Palustrol	3.2699	
39.452	Nerolidol	1.4601	
28.035	β-Chamigrene	1.3231	Terpene
29.732	Cyclohexane, 1-ethenyl-1-methyl-2,4-bis(1-methylethenyl)-, [1S-(1à,2á,4á)]-	1.0102	
30.759	(−)-α-Cedrene	2.179	
32.357	Guaia-10(14),11-diene	2.7849	
33.415	β-Cubebene	4.2486	
34.095	γ-Gurjunene	1.6816	
34.685	γ-Cadinene	1.0292	
34.941	Cadina-1(10),4-diene	2.3372	

^a^: Retention time. ^b^: Twenty-three compounds were identified by comparing the mass spectra and retention times from those of available standards in the Library of the National Institute of Standards and Technology (NIST05). ^c^: Compounds produced in the control were not included in table.

## Data Availability

The raw data supporting the conclusions of this article will be made available by the authors, without undue reservation.

## Supplementary Materials

The following supporting information can be downloaded at: https://www.mdpi.com/article/10.3390/jof8121291/s1, Table S1. Growth characteristics of strain Y1-14 on different culture media; Table S2. Physiological and biochemical characteristics of *Streptomyces* sp. Y1-14; Table S3. Carbon and nitrogen source utilization of *Streptomyces* sp. Y1-14; Figure S1. Determination and analysis of VOCs produced by *Streptomyces* sp. Y1-14. (A) Total ion current chromatograms of VOCs. (B) Antifungal activity against Foc TR4 of different concentrations of Nerolidol; Figure S2. Effects of *Streptomyces* sp. Y1-14 Extracts on Foc TR4 Metabolites. (A) PCA-3D diagram of all samples. (B,C) Heatmap based on the metabolome data of Foc TR4 treated with extracts of *Streptomyces* sp. Y1-14 at 6 h and 12 h. (D,E) HMDB classification diagram of different metabolites in each group.

## References

[1] Li H., Hu C., Xie A., Wu S., Dou T. (2022). Overexpression of *MpbHLH* transcription factor, an encoding ICE1-like protein, enhances Foc TR4-resistance of Cavendish banana. Sci. Hortic..

[2] (2019). Food and Agriculture Organization of the United Nations: FAOSTAT Crops. https://www.fao.org/faostat/en/#data/QC.

[3] Rebouas T.A., de Jesus Rocha A., Cerqueira T.S., Adorno P.R., Barreto R.Q., Ferreira M., Lino L.S.M., Amorim V.B.D., Santos-Serejo J.A.D., Haddad F. (2021). Pre-selection of banana somaclones resistant to *Fusarium oxysporum* f. sp. *cubense*, subtropical race 4. Crop Prot..

[4] Hong S., Jv H., Lu M., Wang B., Ruan Y. (2020). Significant decline in banana Fusarium wilt disease is associated with soil microbiome reconstruction under chilli pepper-banana rotation. Eur. J. Soil Biol..

[5] Tripathi L., Ntui V.O., Tripathi J.N. (2020). CRISPR/Cas9-based genome editing of banana for disease resistance. Curr. Opin. Plant Biol..

[6] Zapata-Sarmientoa D.H., Palacios-Palaa E.F., Rodríguez-Hernándezb A.A., Melchora D.L.M., Rodríguez-Monroya M., Sepúlveda-Jiménez G. (2019). *Trichoderma asperellum,* a potential biological control agent of *Stemphylium vesicarium*, on onion (*Allium cepa* L.). Biol. Control..

[7] Bubici G., Kaushal M., Prigigallo M.I., Gómez-Lama Cabanás C., Mercado-Blanco J. (2019). Biological control agents against Fusarium wilt of banana. Front. Microbiol..

[8] Thangavelu R., Gopi M. (2015). Combined application of native *Trichoderma* isolates possessing multiple functions for the control of Fusarium wilt disease in banana cv. grand naine. Biocontrol Sci. Technol..

[9] Li C., Cheng P., Zheng L., Li Y., Yu G. (2021). Comparative genomics analysis of two banana Fusarium wilt biocontrol endophytes *Bacillus subtilis* R31 and TR21 provides insights into their differences on phytobeneficial trait. Genomics.

[10] Xu Z., Wang M., Du J., Huang T., Chen Y. (2020). Isolation of *Burkholderia* sp. HQB-1, a promising biocontrol bacteria to protect banana against Fusarium wilt through phenazine-1-carboxylic acid secretion. Front. Microbiol..

[11] Bérdy J. (2005). Bioactive microbial metabolites. J. Antibiot..

[12] Bhatti A.A., Haq S., Bhat R.A. (2017). Actinomycetes benefaction role in soil and plant health. Microb. Pathog..

[13] Bu Q.T., Li Y.P., Xie H., Li J.F., Li Y.Q. (2021). Rational engineering strategies for achieving high-yield, high-quality and high-stability of natural product production in actinomycetes. Metab. Eng..

[14] Dede A., Güven K., Ahn N. (2020). Isolation, plant growth-promoting traits, antagonistic effects on clinical and plant pathogenic organisms and identification of actinomycetes from olive rhizosphere. Microb. Pathog..

[15] Shan W., Zhou Y., Liu H., Yu X. (2018). Endophytic actinomycetes from tea plants (*Camellia sinensis*): Isolation, abundance, antimicrobial, and plant-growth-promoting activities. Biomed Res. Int..

[16] Li X., Li K., Zhou D., Zhang M., Qi D., Jing T., Zang X., Qia C., Wang W., Xie J. (2021). Biological control of banana wilt disease caused by *Fusarium oxyspoum* f. sp. *cubense* using *Streptomyces* sp. H4. Biol. Control.

[17] Zhang L., Zhang H., Huang Y., Peng J., Xie J., Wang W. (2021). Isolation and evaluation of rhizosphere actinomycetes with potential application for biocontrolling Fusarium wilt of banana caused by *Fusarium oxysporum* f. sp. cubense tropical race 4. Front. Microbiol..

[18] Wang J., Cai B., Li K., Zhao Y., Li C., Liu S., Xiang D., Zhang L., Xie J., Wang W. (2022). Biological control of *Fusarium oxysporum* f. sp. *cubense* tropical race 4 in banana plantlets using newly isolated *Streptomyces* sp. WHL7 from marine soft coral. Plant Dis..

[19] Ou Y., Penton C.R., Geisen S., Shen Z., Shen Q. (2019). Deciphering underlying drivers of disease suppressiveness against pathogenic *Fusarium oxysporum*. Front. Microbiol..

[20] Williams S.T., Goodfellow M., Alderson G., Wellington E., Sackin M.J. (1983). Numerical classification of *Streptomyces* and related genera. J. Gen. Microbiol..

[21] Wei Y., Zhao Y., Zhou D., Qi D., Wang W. (2020). A newly isolated *Streptomyces* sp. YYS-7 with a broad-spectrum antifungal activity improves the banana plant resistance to *Fusarium oxysporum* f. sp. *cubense* tropical race 4. Front. Microbiol..

[22] Zou N., Zhou D., Chen Y., Lin P., Chen Y., Wang W., Xie J., Wang M. (2021). A novel antifungal actinomycete *Streptomyces* sp. strain h3-2 effectively controls banana Fusarium wilt. Front. Microbiol..

[23] Ahmad M.S., EI-Gendy A.O., Ahmed R.R., Hassan H.M., EI-Kabbany H.M., Merdash A.G. (2017). Exploring the antimicrobial and antitumor potentials of *Streptomyces* sp. AGM12-1 isolated from Egyptian soil. Front. Microbiol..

[24] Qi D., Zou L., Zhou D., Chen Y., Wang W. (2019). Taxonomy and broad-spectrum antifungal activity of *Streptomyces* sp. SCA3-4 isolated from rhizosphere soil of *Opuntia stricta*. Front. Microbiol..

[25] Kumar S., Stecher G., Tamura K. (2016). Mega7: Molecular evolutionary genetics analysis version 7.0 for bigger datasets. Mol. Biol. Evol..

[26] Jing T., Zhou D., Zhang M., Yun T., Qi D., Wei Y., Chen Y., Zang X., Wang W., Xie J. (2020). Newly isolated *Streptomyces* sp. JBS5-6 as a potential biocontrol agent to control banana Fusarium wilt: Genome sequencing and secondary metabolite cluster profiles. Front. Microbiol..

[27] Li X., Jing T., Zhou D., Zhang M., Qi D., Zang X., Zhao Y., Li K., Tang W., Chen Y. (2021). Biocontrol efficacy and possible mechanism of *Streptomyces* sp. H4 against postharvest anthracnose caused by *Colletotrichum fragariae* on strawberry fruit. Postharvest Biol. Technol..

[28] Vanewijk P., Hoekstra J. (1993). Calculation of the EC_50_ and its confidence interval when subtoxic stimulus is present. Ecotoxicol. Environ. Saf..

[29] Chen Y., Zhou D., Qi D., Gao Z., Luo Y. (2017). Growth promotion and disease suppression ability of a *Streptomyces* sp. cb-75 from banana rhizosphere soil. Front. Microbiol..

[30] Dennis C., Webster J. (1971). Antagonistic properties of species-groups of *Trichoderma*: I. Production of non-volatile antibiotics. Trans. Brit. Mycol. Soc..

[31] Rajani P., Rajasekaran C., Vasanthakumari M.M., Olsson S.B., Shaanker R.U. (2021). Inhibition of plant pathogenic fungi by endophytic *Trichoderma* spp. through mycoparasitism and volatile organic compounds. Microbiol. Res..

[32] Francesco A.D., Ugolini L., Lazzeri L., Mari M. (2015). Production of volatile organic compounds by *Aureobasidium pullulans* as a potential mechanism of action against postharvest fruit pathogens. Biol. Control..

[33] Mizutani A., Yukioka H., Tamura H., Miki N., Masuko M., Takeda R. (1995). Respiratory characteristics in *Pyricularia oryzae* exposed to a novel alkoxyiminoacetamide fungicide. Phytopathology.

[34] Wang Z., Wang C., Li F., Li Z., Chen M., Wang Y., Qiao X., Zhang H. (2013). Fumigant activity of volatiles from *Streptomyces alboflavus* TD-1 against *Fusarium moniliforme* Sheldon. J. Microbiol..

[35] Gerber N.N. (1969). A volatile metabolite of actinomycetes, 2-methylisoborneol. J. Antibiot..

[36] Ogura T., Sunairi M., Nakajima M. (2000). 2-methylisoborneol and geosmin, the main sources of soil odor, inhibit the germination of brassicaceae seeds. Soil Sci. Plant Nutr..

[37] Rădulescu M., Jianu C., Lukinich-Gruia A.T., Mioc M., Mioc A., Șoica C., Stana L.G. (2021). Chemical composition, in vitro and in silico antioxidant potential of *Melissa officinalis* subsp. *officinalis* essential oil. Antioxidants.

[38] Dalli M., Azizi S.E., Benouda H., Azghar A., Tahri M., Bouammali B., Maleb A., Gseyra N. (2021). Molecular composition and antibacterial effect of five essential oils extracted from *Nigella sativa* L. seeds against multidrug-resistant bacteria: A comparative study. Evid. Based Complement. Altern. Med..

[39] Schmidt R., Cordovez V., de Boer W., Raaijmakers J., Garbeva P. (2015). Volatile affairs in microbial interactions. ISME J..

[40] Audrain B., Farag M.A., Ryu C.M., Ghigo J.M. (2015). Role of bacterial volatile compounds in bacterial biology. FEMS Microbiol. Rev..

[41] Yang M., Huang C., Xue Y., Li S., Lu L., Wang C. (2018). Biofumigation with volatile organic compounds from *Streptomyces alboflavus* TD-1 and pure chemicals to control *Aspergillus ochraceus*. Ann. Appl. Biol..

[42] Gouda S., Das G., Sen S.K., Shin H.S., Patra J.K. (2016). A treasure house of bioactive compounds of medicinal importance. Front. Microbiol..

[43] Chang P.C., Liu S.C., Ho M.C., Huang T.W., Huang C.H. (2022). A soil-isolated *Streptomyces spororaveus* species produces a high-molecular-weight antibiotic AF1 against fungi and Gram-positive bacteria. Antibiotics.

[44] Kopp D., Sunna A. (2020). Alternative carbohydrate pathways-enzymes, functions and engineering. Crit. Rev. Biotechnol..

[45] Noor E., Eden E., Milo R., Alon U. (2010). Central carbon metabolism as a minimal biochemical walk between precursors for biomass and energy. Mol Cell..

[46] Nelson D.L., Cox M.M. (2005). Lehninger Principles of Biochemistry.

[47] Flamholz A., Noor E., Bar-Even A., Liebermeister W., Milo R. (2013). Glycolytic strategy as a tradeoff between energy yield and protein cost. Proc. Natl. Acad. Sci. USA.

[48] Bertels L.K., Fernández M.L., Heinisch J.J. (2021). The pentose phosphate pathway in yeasts-more than a poor cousin of glycolysis. Biomolecules.

[49] Wood T. (1985). The Pentose Phosphate Pathway.

[50] Sprenger G.A. (1995). Genetics of pentose-phosphate pathway enzymes of *Escherichia coli* K-12. Arch. Microbiol..

[51] Yin X., Li J., Shin H.D., Du G., Liu L., Chen J. (2015). Metabolic engineering in the biotechnological production of organic acids in the tricarboxylic acid cycle of microorganisms: Advances and prospects. Biotechnol. Adv..

[52] Fernie A.R., Carrari F., Sweetlove L.J. (2004). Respiratory metabolism: Glycolysis, the TCA cycle and mitochondrial electron transport. Curr. Opin. Plant Biol..

[53] Eniafe J., Jiang S. (2021). The functional roles of TCA cycle metabolites in cancer. Oncogene.

[54] Jia Y., Wong D.C., Sweetman C., Bruning J.B., Ford C.M. (2015). New insights into the evolutionary history of plant sorbitol dehydrogenase. BMC Plant Biol..

[55] Hua X., Liu W., Su Y., Liu X., Liu J., Liu N., Wang G., Jiao X., Fan X., Xue C. (2020). Studies on the novel pyridine sulfide containing SDH based heterocyclic amide fungicide. Pest Manag. Sci..

[56] Venkat S., Gregory C., Sturges J., Gan Q., Fan C. (2017). Studying the lysine acetylation of malate dehydrogenase. J. Mol. Biol..

