# Supplementary material for: Antifungal Mechanism of Metabolites from Newly Isolated Streptomyces sp. Y1-14 against Banana Fusarium Wilt Disease Using Metabolomics
Source: J Fungi (Basel). 2022 Dec 9;8(12):1291. doi: 10.3390/jof8121291 (PMC9784640; doi:10.3390/jof8121291)
Supplement: Supplementary file 1 [file jof-08-01291-s001.zip › jof-1988321-supplementary.pdf]

SUPPLEMENTARY MATERIAL

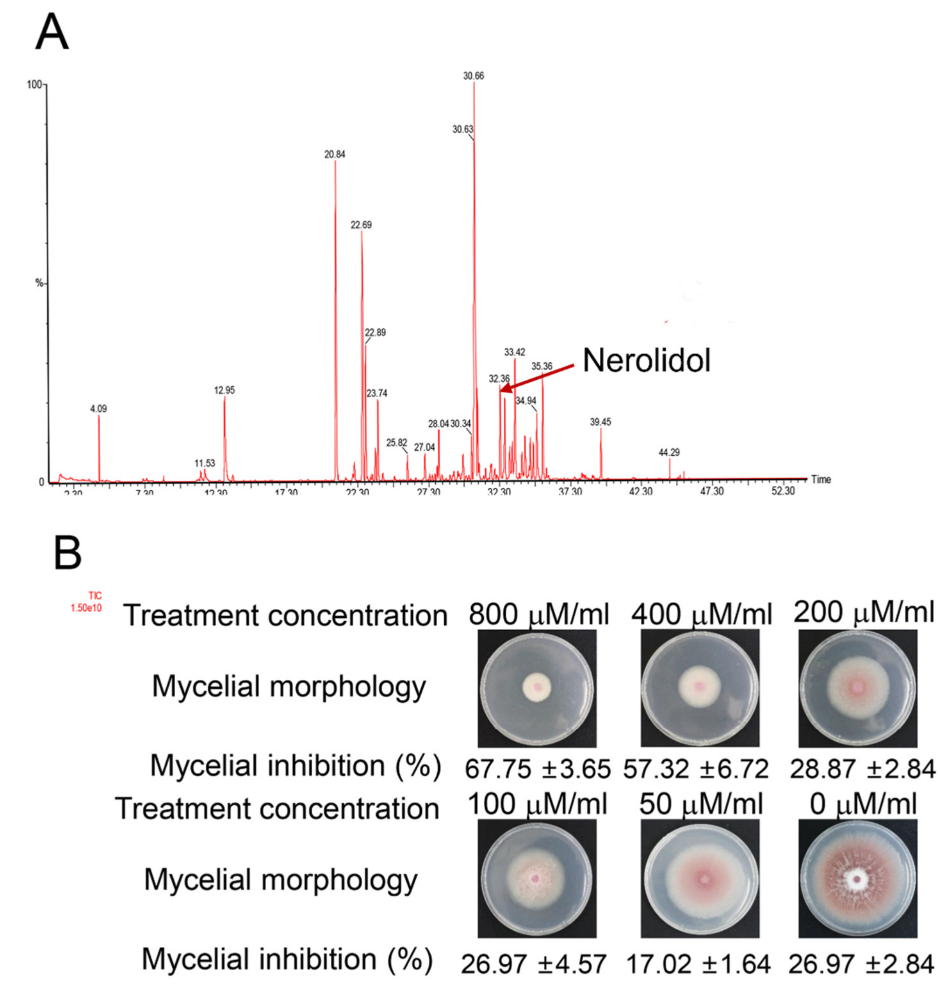

Figure S1 | Determination and analysis of VOCs of strain Y1-14 and validation of active components.

(A) Total ion current chromatograms of VOCs of strain Y1-14. The red arrow represents nerolidol.

(B) Antifungal activity against Foc TR4 of different concentrations of Nerolidol.

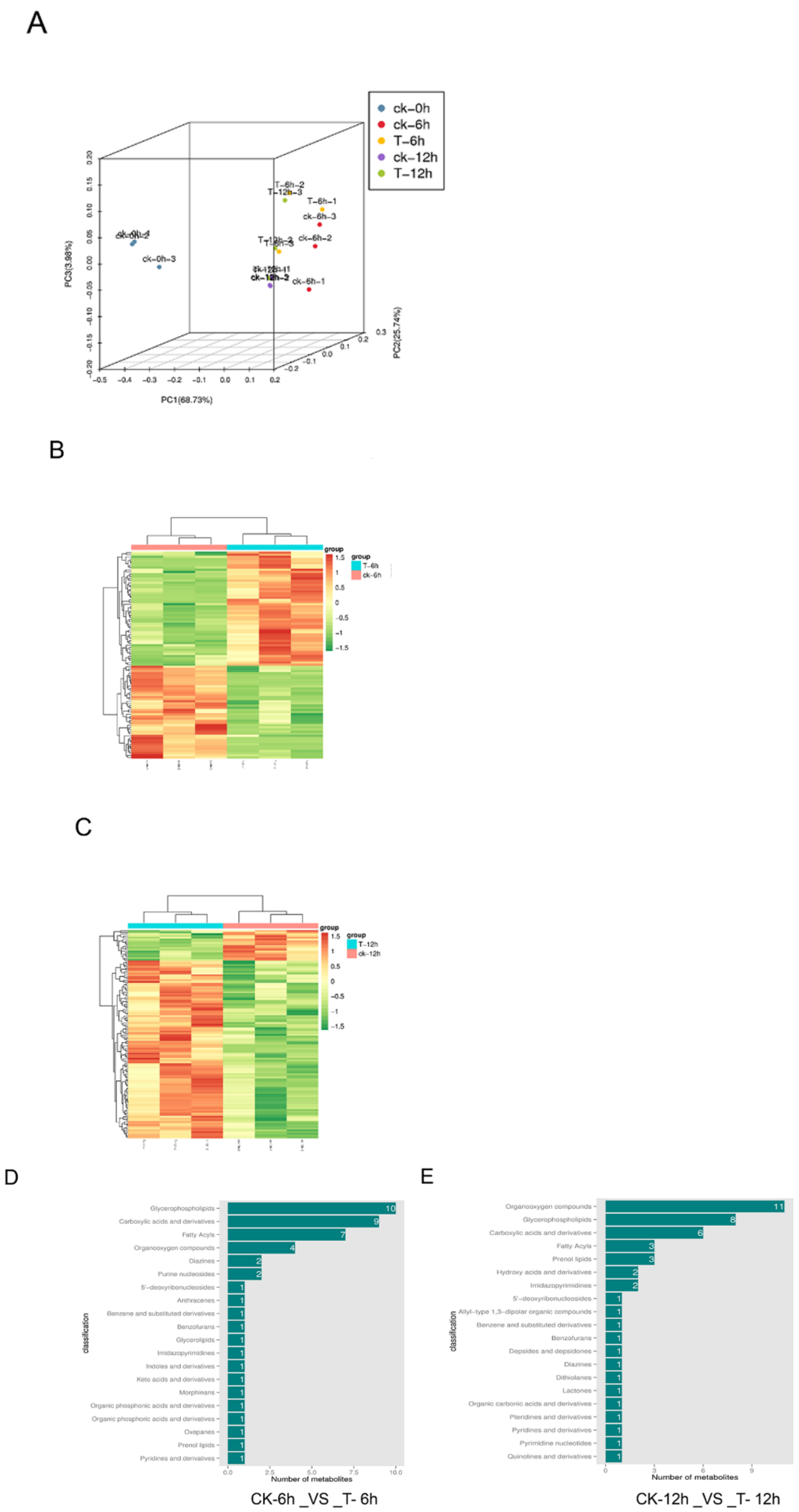
A
ck–0h
ck–6h
T–6h
ck–12h
T–12h
PC1(68.73%)
PC2(25.74%)
PC3(3.98%)
B
group
T–6h
ck–6h
C
group
T–12h
ck–12h
D
Glycerophospholipids
Carboxylic acids and derivatives
Fatty Acyls
Organooxygen compounds
Diazines
Purine nucleosides
5'–deoxyribonucleosides
Anthracenes
Benzene and substituted derivatives
Benzofurans
Glycerolipids
Imidazopyrimidines
Indoles and derivatives
Keto acids and derivatives
Morphinans
Organic phosphonic acids and derivatives
Organic phosphoric acids and derivatives
Oxepanes
Prenol lipids
Pyridines and derivatives
classification
Number of metabolites
CK-6h _VS _T- 6h
E
Organooxygen compounds
Glycerophospholipids
Carboxylic acids and derivatives
Fatty Acyls
Prenol lipids
Hydroxy acids and derivatives
Imidazopyrimidines
5'–deoxyribonucleosides
Allyl–type 1,3–dipolar organic compounds
Benzene and substituted derivatives
Benzofurans
Depsides and depsidones
Diazines
Dithiolanes
Lactones
Organic carbonic acids and derivatives
Pteridines and derivatives
Pyridines and derivatives
Pyrimidine nucleotides
Quinolines and derivatives
classification
Number of metabolites
CK-12h _VS _T- 12h

Figure S2 | Effects of strain Y1-14 Extracts on Foc TR4 Metabolites. (A) PCA-3D diagram of all samples. (B-C) Heatmap based on the metabolome data of Foc TR4 treated with extracts of Y1-14 and untreated at 6h and 12h. (D-E) HMDB classification diagram of different metabolites in each group.

Table S1. Growth characteristics of strain Y1-14 on different solid culture media

**Medium**	**Aerial mycelium**	**Vegetative mycelium**	**Soluble pigment**	**Growth**	**Colony**
ISP3	Mint cream	White	None	++	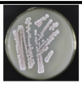
ISP4	Mint cream	Burlywood	Grey	++	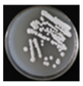
ISP5	Beige	Mint cream	None	++	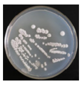
ISP6	Mint cream	Wheat	Pink	+	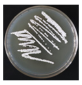
ISP7	Mint cream	Burlywood	None	++	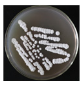
PDA	Mint cream	lemon yellow	None	++	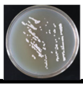

+ good growth; ++ better growth; +++ best growth

Table S2. Physiological and biochemical characteristics of strain Y1-14

**Characteristics**	**Results**
pH range for growth	4-10
NaCl tolerance for growth (%)	0-5
H_2S production	+
Milk peptonization	+
Starch hydrolysis	+
Cellulose hydrolysis	+
Gelatin liquefaction	-
Nitrate reduction	-

+, Positive reaction; −, Negative reaction

Table S3. Carbon and nitrogen utilization characteristics of strain Y1-14

**Nitrogen Source Utiliziton**		**Carbon Source Utilizition**	
Nitrogen Source	Result	Carbon Source	Result
L-arginine	+	Salicylicin	-
Tyrosine	+	D-Cellobiose	+
Aspartamide	+	Raffinose	+
Thiine	+	D-Galactose	+
ammonium nitrate	+	D-Glucose	+
L-Glutamic acid	-	Sorbitol	+
L (+)-cysteine	+	D-Sorbitol	+
Phenylalanine	+	D-Fructose	+
D-Mannitol	+	Soluble starch	+
Inositol	-	Trisaccharide	+
Rhamnose	+	Anhydrous amino acid	-
Sodium propionate	+	Ribose	+

+, Positive reaction; −, Negative reaction
